# Why the Country Practitioner Should Not Do Major Surgery*Read before the Fifth and Sixth District Medical Society, September 8 and 9, 1914, at Corpus Christi, Texas.

**Published:** 1914-11

**Authors:** R. C. Youngblood

**Affiliations:** Falls City, Texas


For Texas Medical Journal.

### Why the Country Practitioner Should Not Do Major Surgery.*

BY R. C. YOUNGBLOOD, M. D., FALLS CITY, TEXAS.

Being a staunch believer in the much commercialized words, "safety first," I unhesitatingly say that no country physician should do major surgery. Neither do I believe that any pseudo-surgeon should handle the vital organs of men. To explain, recently a distinguished physician after visiting twenty-four States in the Union made the following statement: "In a certain city, where there were more than a hundred practitioners of medicine, 43 per cent were doing major surgery. Practically all these 'surgeons' do all the practice they can get, and the principal work of the few really capable surgeons there consists in patching up botched surgical jobs. These are, perhaps, hard charges to make, but they are absolutely true." Thus we see that this surgical craze has not only infested the *one city,* but many, the country as well, with actual harm resulting from such practice.

I believe that major surgery should be confided to those that make a specialty of surgery. For a country practitioner to persist in doing major surgery, to me is an irrational act, and is not warranted. I fully realize the dilemma in which I have placed myself by making the above statement, for I know some eminent surgeons who are doing general practice, and, on the other hand, some good practitioners doing surgery. I believe this is wrong; their interest should be mutual, and each should respect the other's specialty. This condition, more than any other, is the result of so many surgical failures.

For an illustration: Suppose the country practitioner does a major operation; subsequently he is called away in the country to see a lady in confinement. What is he to do? He has promised his services in advance; they expect him to take the case regardless of his previous engagement. The question is, can he do it and treat the former party with the same consideration? Do you not think it would be criminal in case a complication should arise in the surgical case while the physician is away? Assistance cannot be obtained as easily in the country as in the city.

Primarily, we will notice some of the objections that the coun-

*Read before the Fifth and Sixth District Medical Society, September 8 and 9, 1914, at Corpus Christi, Texas.

try surgeon has in sending his surgical cases to the city surgeon. Secondarily, to intrench his own opinion in the belief that it is right for the country physician to do major surgery.

First objection: To save time by operating in the country. Time is valuable, that is true, and more especially if one has a case with strangulated hernia, or where one wishes to do a drain operation; or in case of an injury where it becomes necessary to operate at once. But we must remember that the country physician has not everything prepared to do an operation; it requires time to prepare for a major operation and secure assistance, even if one is so fortunate to get it without delay, which is often the case in the country. Owing to rapid transit, excellent service and a goodly number of modern equipped hospitals throughout the country, to save time, would mean to go to some nearby hospital, where everything is in readiness.

Second objection: The expense to the patient. When the country surgeon secures his assistants, consisting of anesthetist, assistant operator, two nurses, and to retain one on the case for two or three weeks; charging a reasonable fee for his own services, will in the end amount to as much if not exceed the city hospital expenses.

Third objection: Developing a surgical sense by comparing the symptoms of the disease with the pathological condition after the surgical finding. I grant this is true; it is a great way to enhance one's knowledge in the practice of medicine and surgery. If the attending physician is sufficiently interested, he may accompany the patient to the hospital, and gather this knowledge without subjecting his patient to any hazardous risk.

Fourth objection: To prevent the city surgeon from receiving all the reputation. It detracts not from the country doctor's reputation to diagnose a surgical condition and deliver his patient over to a competent surgeon. On the other hand, it strengthens the confidence of the laity in the doctor's medical skill.

The country physician should not do major surgery, because he has not a modern well equipped hospital. He has not the well-trained and well-organized assistants. To do effective surgical work requires organization. Anything short of this will not do. The country physician has none of these. He has to content himself with some farm house or office; the former is bad and the latter is worse. How seldom do we see a real nice, clean, tidy country office. It has been a wonder to me that some doctors do not die with the bubonic plague. To operate in the residence with the generous public as an audience is by no means gratifying to a surgeon who is anticipating the best results. I grant that some successful surgery has been done in the country under the most adverse circumstances, and I give them full credit, for it is justly deserving. I note that the practicing surgeon of the country takes full measure of the fact, and refers to the eminent surgeon that hailed from there. After considering the facts, I believe there is a way to do better surgery, and that way is safety first way.

